# SARS-CoV-2: Challenges in Reconverting Diagnostic Laboratories to Combat the Pandemic

**DOI:** 10.1128/spectrum.01477-22

**Published:** 2022-10-31

**Authors:** Hugo Alberto Barrera Saldaña, Carolina Rivera Santiago, Raúl Rodríguez Palacios

**Affiliations:** a Columbia Comercial, SA de CV, División Columbia Biotec, Tlalpan, Mexico City, México; b Vitagénesis, SA de CV, and Innbiogem SC at LANSEIDI-CONACyT, Monterrey, Nuevo León, México; c Universidad Autónoma de Nuevo León, School of Medicine, San Nicolás de los Garza, Nuevo León, México; d Universidad Autónoma de Nuevo León, School of Biological Sciences, San Nicolás de los Garza, Nuevo León, México; University of Mississippi Medical Center

**Keywords:** SARS-CoV-2 diagnosis, laboratory techniques, commercial RT-PCR kits, RT-PCR

## Abstract

Coronavirus disease 2019 (COVID-19) was first detected in Mexico in February 2020. Even though health authorities did not perceive then the value of viral detection tests, we anticipated the demand for them. We set up to develop an expeditious severe acute respiratory syndrome coronavirus 2 (SARS-CoV-2) molecular diagnostic service through the implementation of standardized protocols for biospecimen sampling, transportation, biobanking, preanalytical validation, and nucleic acids (NA) testing (NAT). Nasopharyngeal and oropharyngeal swabs collected in a special transportation medium were the biospecimens from which NAs were purified either manually or automatically. Viral RNA genome presence was determined using commercial SARS-CoV-2 detection kits (based on reverse transcription coupled with real-time PCR [RT-PCR]). Improvements in laboratory processing speed and reliability resulted from semi-automatizing laboratory processes and adopting a quality control/quality assurance system (QC/QA), respectively. NAs that were purified, either manually or automatically, were validated by preanalytical spectrophotometric characterization. Automated purification was less prone to contamination and reduced the processing time. The following six RT-PCR kits were evaluated for their convenience, specificity, sensitivity, time consumption, and required materials (in order, starting with the kit with the best results): RIDA gene and Viasure (tied), Vircell, LightMix, 1copy, and Logix Smart. Redesigning the laboratories’ working areas, equipment, fluxes of personnel and material, and personnel skills, and overemphasizing biosafety safeguards were major challenges encountered in the middle of the sanitary crisis. Adopting a QC/QA system, utilizing automatization processes, and working closely with health authorities were key factors in our success.

**IMPORTANCE** Rearranging our diagnostic laboratories to improve the fight against a new unexpected, unpredictable, and sudden public health threat demanded that we move quickly to redesign not only the laboratory processes but also the distribution of space, personnel activities, and fluxes of material coming in and out. We also had to work closely with governmental health authorities to gain their trust in our technical competence. Gaining the confidence of the clients, i.e., mainly individuals, the human resource departments of factories and corporations sending employees for testing, and medical institutions, and implementing as much automatization as possible of processes, in which only officially approved reagents (for extraction and analysis of NA) were used to generate opportune trustable testing results, were key factors. Our laboratories have gathered a considerable amount of experience and significant number of solutions, considering our geographic contexts alongside this continuously morphing pandemic, validating many techniques that might help other laboratories find a better and more precise workflow.

## INTRODUCTION

Coronavirus disease 2019 (COVID-19) was declared a pandemic on 11 March 2020 by the World Health Organization (WHO), after its rapid spread around the world (1). The disease-causing agent was discovered to be a novel coronavirus causing severe acute respiratory syndrome (SARS), named coronavirus 2 (SARS-CoV-2) by the Study Group of the International Committee on Taxonomy of Viruses, based on the phylogeny and taxonomy of the newly isolated virus (2–4). It is worth mentioning that SARS-CoV-2 is not a descendant of the virus that was known to cause a previous SARS outbreak (SARS-CoV); rather, the name was obtained based on the established practice of naming viruses and because it shares a high sequence identity with the latter (5).

The onset of the disease was first identified in the respiratory tracts of pneumonia patients in December 2019 in Wuhan, China (6, 7). Due to the huge impact on public health and the global economy that the pandemic has had and is still imposing (77,815.15 cumulative confirmed COVID-19 cases per million people, including 826.62 cumulative confirmed COVID-19 deaths per million people reported by Our World In Data as of 26 September 2022) (8), health and research institutions around the world have made and continue to exert great efforts to understand the course of the disease, so that its behavior can be predicted, the evolving clinical manifestation can be understood, and means for treating it are identified. These efforts have been made while relying on pathological and therapeutic evidence from other viruses of the same family that have caused epidemics in humans, such as the abovementioned SARS-CoV and Middle East respiratory syndrome coronavirus (MERS-CoV) (4, 9).

COVID-19 spreads more quickly than flu pandemics, with early flu-like symptoms comprising pulmonary affectation, in many cases with a late hyperinflammatory response resulting in multisystem damage and consequently, the need for supplemental oxygenation, hospitalization, and intubation, increasing the death rate greatly. Severity and lethality were soon associated with preexisting comorbidities, such as obesity, diabetes, and hypertension, among others (10).

Weeks after identification of the virus, its approximately 30,000-nucleotide RNA genome was sequenced, and a few months later, diagnostic tests were designed from the sequence. Likewise, realizing the immunodominant role of the spike (S) protein present in the envelope of the virions, a myriad of vaccines to expose the population to it or its coding sequences (as DNA and—without precedence—as RNA) were designed, and clinical trials were initiated. Progress in molecular biology, biotechnology, preclinical validations, and clinical accelerated trials rendered the first vaccines in an amazing record time. Vaccines based on recombinant proteins, engineered viral vectors, and surprisingly enough, mRNA-based gene therapy reached emergency approval; an S-coding mRNA jab from Pfizer was applied outside clinical trials a year later, marking both the dawn of the mRNA therapeutics era and the start of global vaccination campaigns by governments around the world (11).

It has been over 2 years since the first method emerged for detecting the SARS-CoV-2 viral RNA genome, relying on reverse transcription (RT), followed by amplification of specific viral gene sequences in the complementary DNA (cDNA) by the semiquantitative variant (real time) of the PCR (RT-PCR). This approach was soon to be the gold standard for SARS-CoV-2 diagnostic testing (12). Nucleic acid tests (NATs) are considered the most suitable tools for reliable diagnosis of infectious agents, and several commercial NAT kits have been developed for COVID-19. These kits are accompanied by kits for the purification of NAs (including the RNA genome of the virus) from putatively infected cells, as the substrate for RT-PCR kits (13, 14). The viral genes commonly addressed include those coding for the nucleocapsid (N), envelope (E), RNA-dependent RNA polymerase (RdRP), glycoprotein surface proteins (S), and several open reading frames (ORFs) of the viral genome; within these, relatively highly conserved regions are the preferred targets (15). The WHO recommends searching simultaneously for two different viral gene targets for more reliable detection, as well as including controls. For example, to assess the quality of the sample, the cellular gene for RNase P is used to exclude the presence of inhibitors of the PCR, while to verify the effectiveness of the reverse transcription reaction to which the RNA in the sample is subjected, a synthetic RNA is added to the RT-PCR master mix (16–18).

However, the exponential spread of the disease, the urgency of screening during the earlier stages of infected cases, which are inadvertent sources of contagious disease, and the aggravation due to the shortage of NAT diagnostic kits led to the development of additional techniques for the direct diagnosis of COVID-19 (19). Instead of targeting the viral genes, these techniques rely on antibodies that target viral antigens, generally the S protein but also the N protein, bound in lateral flow immunoassay devices (which are qualitative chromatographic tests that render either positive or negative results). They provide rapid results (in less than 30 min) to screen for the presence of the virus in the naso- or oropharyngeal exudate swabs of individuals with suspicious symptoms (16, 20, 21).

Disease diagnosis and evolution are also determined clinically and through imaging studies, such as a thoracic X ray. Moreover, to monitor the progress of the disease, the antibody response is evaluated by detecting immunoglobulin M (IgM; the first Ig type through which the immune system responds to antigens, in a matter of days) and immunoglobulin G (IgG; Ig that replaces IgM as the immune response matures a couple of weeks after infection) specific for said viral antigens (19, 20). Besides serum or plasma, they work also with saliva, sputum, and other biological fluids, although allegedly less effectively for the latter three (22). Given the apparent relatively fast decline in the humoral protection achieved with a complete vaccination regimen, a booster has been recommended. Quantitative follow-up of anti-S (typical due to vaccination, although not discriminated from infection) or anti-N (a reflection of past infection) IgG levels in serum by enzyme-linked immunosorbent assay (ELISA) is suggested to guide the frequency of eventual further booster applications (16, 22).

Early, reliable, and accurate diagnosis is crucial to provide medical care to infected individuals, as well as to prevent the spread to others. However, many laboratories with no or little previous experience in PCR jumped into diagnosing COVID-19, thinking that using commercial kits made them infallible. False-negative result rates are a serious concern, since they have been reported to fluctuate from 1% to 30% (23). Offering diagnostic services without previous training or with little experience handling NATs can instead backfire, spreading the virus in the community; on the other hand, false-positive results from such laboratories lead to unnecessary treatment, a negative psychological impact on the patient, and even aggravation of the already serious impact on societal and familial economies.

One of the main reasons to adapt our molecular diagnostic laboratories to face COVID-19 was to help enterprises (both Monterrey [where Vitagenesis is located] and Mexico City [location of Columbia Biotec] are national places of the highest concentration of industries) in their efforts to guarantee a safe working environment for employees. By opportunely detecting the virus, we pledged to undertake the timely discovery of otherwise inadvertent cases that represent a serious risk to a company’s workforce and workers’ families. While the adoption of sanitary measures and monitoring of infection signs have proven to be efficacious, combining them with laboratory screening for the virus is the best that can be done to minimize the inevitable societal and economic impact of this infamous pandemic. Such laboratory information in the hands of company medical directors becomes strategic for implementing the most efficacious sanitary defenses against this fearsome enemy. Here is an account of our transition from being mainly cancer diagnosis laboratories to becoming COVID-19 government-authorized private reference laboratories.

## RESULTS

### Gaining official authorization to perform SARS-CoV-2 laboratory diagnosis.

To obtain the approval of InDRE, the government authority responsible for granting permission to private laboratories to offer diagnostic services to the public, our laboratories first submitted the results obtained with the RT-PCR kit controls, including serial dilutions up to 10^−5^. Once we passed, we then submitted the first set of real samples (negative and positive cases) analyzed in our laboratories. InDRE’s diagnostic lab confirmed our results, and both of our laboratories were given the green light to openly offer SARS-CoV-2 detection services to the public.

### Sample handling and validation.

Several studies have shown that there is no significant difference between nasopharyngeal and oropharyngeal sampling for diagnosis of the virus. Although InDRE recommends collecting both types of swabs from everyone, just using one type of swab instead of the two seemed not to affect the results of our tests; however, it significantly reduced consumption of the collection materials, which were always scarce during the critical periods of the pandemic. So, we routinely relied mostly, but not uniquely, on nasopharyngeal swab sampling of both nostrils with the same swab.

Transportation media (both laboratory-made viral transport medium [VTM] and commercial universal transport medium [UTM]) also became hard to get from vendors due to the demand. The solution, found through intense searching and research, came from the webpage of the Indiana University Clinical Virology health laboratories; we obtained a recipe for preparing an alternative to the products available on the market. The formula consists basically of Hanks’ balanced salt solution, 5% inactivated fetal bovine serum, 50 mg/mL gentamicin (a broad-spectrum aminoglycoside antibiotic), and 250 μg/mL amphotericin B (an antibiotic and antifungal).

For both the safety of the operators and avoidance of false positives, controlling the risk of contagion and contamination was of the greatest importance. Thus, we made sure that personnel wore adequate protection vestments (shoe covers, lab coats, disposable surgical gowns, latex gloves, KN95 or surgical masks, face shields, and head covers), constantly decontaminated the laboratory equipment entering into contact with samples, and sanitized the working areas, both physically (20 min UV irradiation at the beginning, middle, and end of daily tasks) and chemically (spraying 1% sodium hypochlorite and 70% ethanol) nature. Also, all samples to be processed were treated as potentially dangerous, as we did not know their results; thus, special attention was given to proper pipetting and disposal of all material that entered into contact with the potential viral sources.

Realizing that the biggest risk of contamination occurs in the biosafety cabinet, where NA purification from biospecimens is performed, in addition to the abovementioned disinfection protocol, we also maintained an appropriate spatial distribution of the racks holding the samples (the greater the number of samples, the greater their separation) and kept all tubes closed, except those being processed. And to reduce the risk of contamination while opening and closing reagent tubes, we used absorbent material such as disposable paper towels soaked with ethanol and requested that operators constantly disinfect their gloves.

It was particularly critical to control the risk of contamination during NA purification in the biosafety cabinet. Thus, it was decontaminated each morning by spraying onto its surface an Extran detergent solution (catalog no. MX1400005004; Sigma-Aldrich, Mexico City, Mexico), which was left to stand for 30 min, followed by washing the cabinet with distilled water, drying it with disposable paper towels, and another round of disinfection with the abovementioned sodium hypochlorite and ethanol solution. Finally, the cabinet was subjected to an automatic cycle of UV light for 20 min by closing its front window. This cleaning protocol was conducted before the first purification of each day. After the cabinet was completely cleaned both physically and chemically, at the end of the day, once a week (daily if contamination was suspected), an additional reagent to eliminate traces of RNA, called RNase AWAY (catalog no. 7002; Molecular BioProducts, CTR Scientific, Mexico City, Mexico), was distributed onto all internal surfaces of the cabinet and left overnight.

### Nucleic acid purification method selection.

**FIG 1:**
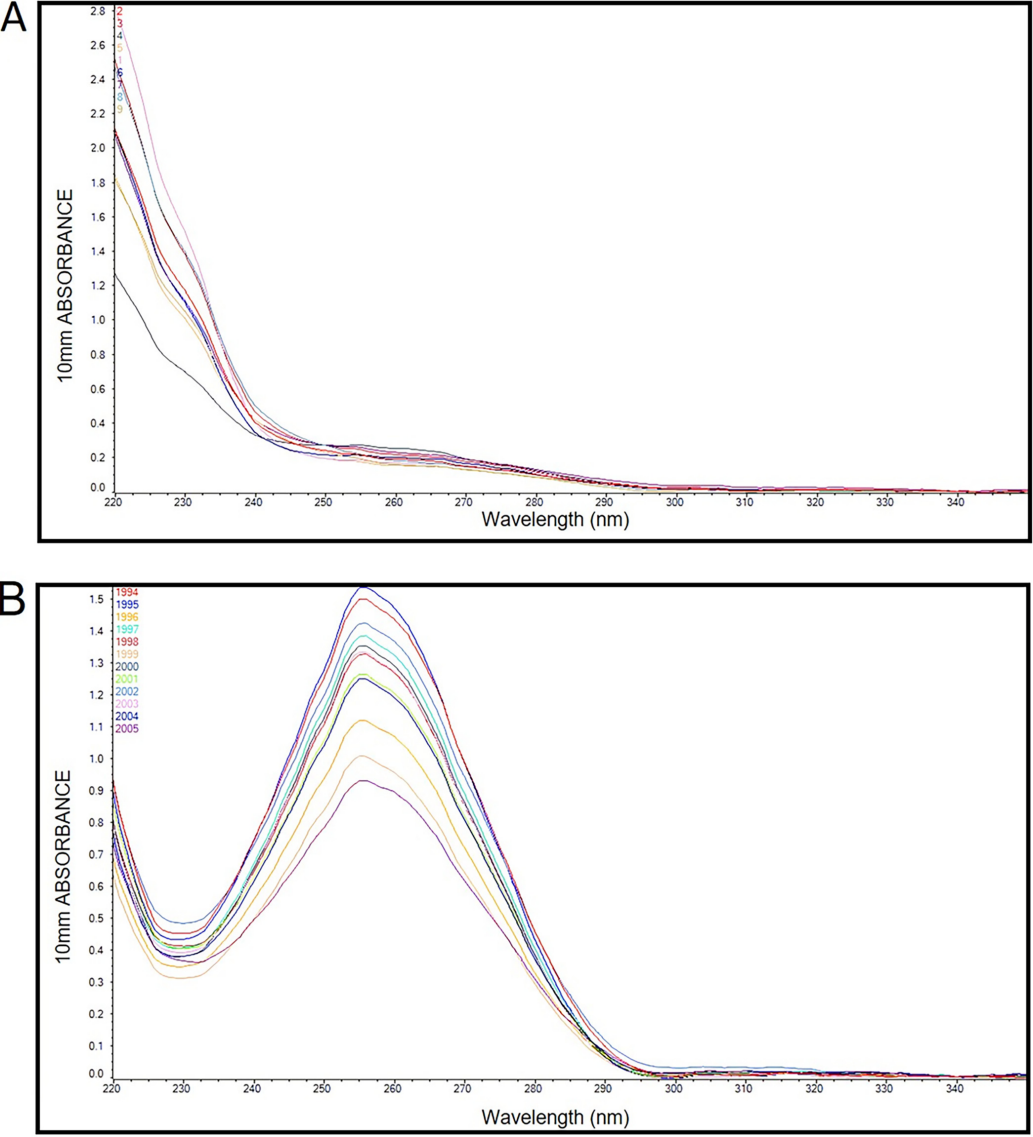
UV spectrum profiles of NA isolated using the automatic method (A) and the manual procedure (B). The *y* axis represents the relative absorbance units, and the *x* axis represents the wavelength.

Nucleic acids from biospecimens (naso- and oropharyngeal swabs) were purified either manually (Qiagen QIAamp viral RNA kit or Daan Gene kit) or automatically (Daan Gene Smart 32 kit). In the manual procedure, the aliquot of transportation medium (VTM or UTM) with the swab resuspended in it was subjected to cell and viral particle lysis and then passed through a column harboring a silica matrix to trap NAs. Undesirable debris and biomolecules trapped in the column were washed away, and the bound NAs were recovered. The automated purification process integrated all these steps, replacing the silica matrix of the columns with silica-lined magnetic beads and using magnetism to recover them after the washing step. We used the manual kit when fewer than 16 samples were ready to be processed and the automatic one when multiples of this number of samples had accumulated for processing. In both cases, we routinely analyzed the rendered NAs by spectrophotometry (typically using the NanoDrop instrument) to both quantify the yield and assess the quality (260 nm/280 nm and 260 nm/230 nm ratios; when possible, UV spectrum scanning). Comparative preanalytical results of the manual versus automatic RNA extraction methods are shown in Table 1, while their typical UV profiles are depicted in Fig. 1. Both methods showed statistically significant differences in their qualitative and quantitative assessments.

**TABLE 1:** Yield and purity of NA samples using the manual versus automated isolation methods^*a*^

Metric	Median (IQR)^*b*^		*P* value
	Manual method	Automated method	
Yield^*c*^ (ng)	1,392 (894–2,190)	880 (536–1,486)	<0.001
Purity (260 nm/280 nm ratio)	2.580 (2.243–2.918)	1.920 (1.690–2.080)	<0.001
Purity (260 nm/230 nm ratio)	2.405 (1.900–2.900)	0.250 (0.170–0.360)	<0.001

a Manual samples, *n* = 968; automated samples, *n* = 1,016.

b IQR, interquartile range.

c Yield indicates the concentration of isolated nucleic acids multiplied by their dilution volumes.

At first glance, we can see that there is a clear difference between the UV spectrum profiles obtained from the automatic and manual purification methods. The atypical profile of the former differs substantially from the typical one of the latter, but this is as expected according to the manufacturer’s data (see Discussion). Typically, manual purification renders more and supposedly cleaner NAs, but the automatic method (which reduced the risk of cross-contamination between samples and was faster) performed equally well in the RT-PCR analysis.

### RT-PCR kit selection.

Since April to May 2020, when our laboratories received the green light from InDRE for our SARS-CoV-2 diagnostic service, we have processed over 50,000 naso- and oropharyngeal swab samples. Since then, the number of diagnostic kits has grown, as have their sensitivity (scoring samples positive only if the virus is present) and specificity (scoring samples negative if the virus is absent even if a virus other than SARS-CoV-2 is present). In addition to these attributes, our comparative evaluation of the kits took into consideration the usefulness of their controls, time and material consumption, and convenience (mono- versus multiplex, whether it required low-temperature conservation) and resulted in the following preferences: (i) RIDA or Viasure, (ii) Vircell, (iii) LightMix, (iv) 1copy, and (v) Logix Smart (see Table 2). It is noteworthy that the RIDA kit came highly recommended in a comparative study carried out by the Dutch Ministry of Health (24).

**TABLE 2:** Comparison of the key features of the RT-PCR kits evaluated

Kit name	RIDA	Viasure	Vircell	LightMix	1 copy	Logix Smart
No. of reactions per sample	1	1	2	3	1	1
Gene SARS-CoV-2 target	E	N, Orf1ab	N, E	N, E, and RdRP	E, RdRP	RdRP
Type of control(s)^*a*^	Exo	Exo	Exo, endo	Exo	Endo	Endo
PCR time (min)	78	104	86	80	80	86
Cutoff (*C_T_*)^*b*^	<45	<40	<40	<39	<40	<45
RNA sample vol (μL)	5	10	10	30	5	5
Ready to use (no mix preparation needed)?	Yes	Yes	No	No	No	No
Preservation (°C)	−20	RT^*c*^	−20, −70	−20	−20	−20
No. of pipette tips spent per reaction	2	2	10	21	10	2
Background noise	None	Medium	None	High	Medium	Low

a Endo, endogenous control, such as the cellular RNase P gene. Exo, exogenous control, such as the addition of artificial RNA.

b *C_T_*, cycle threshold value.

c RT, room temperature.

Both the analytical performance (especially the sensitivity) and the convenience of processing numerous parallel samples in a short period were the key parameters that induced our laboratories to adopt either the RIDA or Viasure kits. They are particularly useful in cases with low viral loads, which even if asymptomatic can be contagious to vulnerable subjects among their families, communities, and work colleagues. Although we alternated between the RIDA and Viasure RT-PCR kits, the Vircell kit was also an option because of the convenience of its controls, especially when the viral load of work was not high, given the extra time needed to assemble the reactions and the materials demanded.

**FIG 2:**
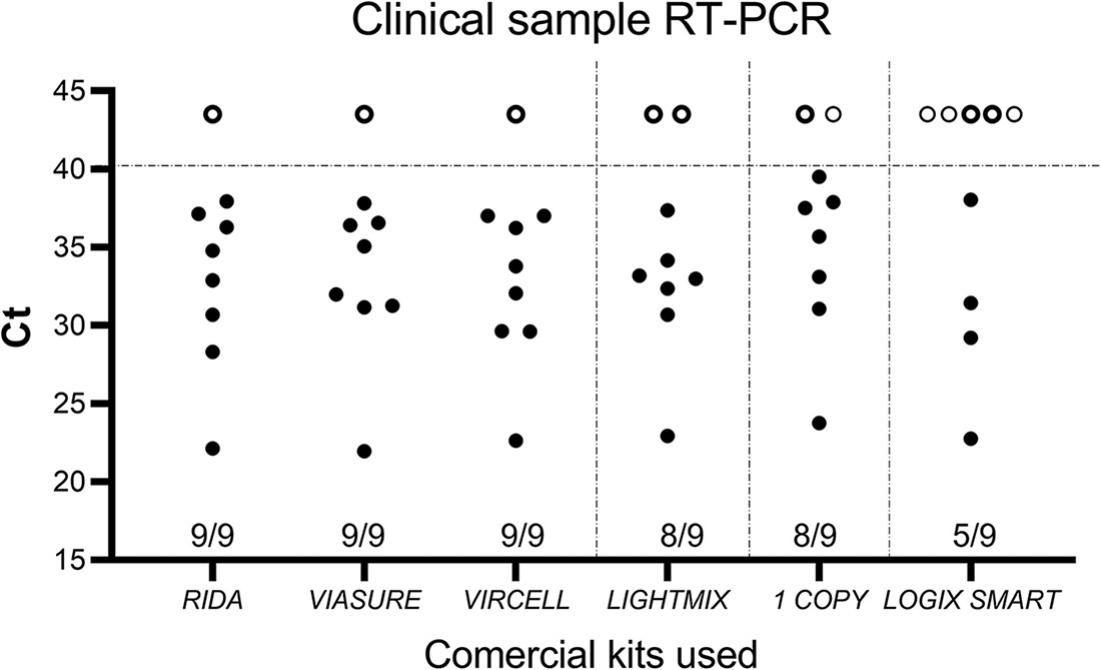
Variations in the detection rate and *C_T_* values in the six kits used. Nine samples (eight positives and one negative) were subjected to the various RT-PCR assays, according to the respective manufacturer’s instructions for use. The solid dots in the graph indicate *C_T_* values obtained for all clinical samples (*n* = 9) using all RT-PCR assays. The open circles above the dotted line are negative, for data purposes indicated by a *C_T_* value of 43.5. The results for the negative sample and the positive one displaying the highest viral load were concordant in all kits evaluated.

In Fig. 2, we show the results from our comparison of the six kits. We observed that there was no discrepancy in the results of the samples with high viral loads. However, at low viral loads, only the Vircell, Viasure, and RIDA gene kits showed concordant results. If we add the convenience of assembling the PCR (see Table 2), the RIDA gene kit is the most convenient and least time-consuming of the three.

It is important to note that the Vircell kit has both endogenous and exogenous controls that are important for the validity of the tests. However, the investment in time and material for its use, as already mentioned, is higher than that required by the RIDA or the Viasure kits.

An additional experiment was carried out to verify the effectiveness of the RIDA and Logix Smart kits, which are similar in terms of the use of inputs and handling time; the results are shown in Table 3. As seen in Fig. 2, here again, the RIDA kit (18/20) surpassed the Logix Smart kit (15/20) in detecting positive cases. It should be noted that the concordant positive results for both kits rendered similar cycle threshold (*C_T_*) values.

**TABLE 3:** Comparison of results from the Logix Smart and RIDA RT-PCR kits

Sample ID	*C_T_* ^*a*^	
	RIDA	Logix Smart
a	ND	ND
b	36.30	ND
c	37.93	38.05
d	37.13	ND
e	28.32	29.22
f	32.88	ND
g	22.13	22.77
h	30.68	31.44
i	34.80	ND
j	36.51	35.68
k	ND	34.7
l	16.48	16.02
m	38.09	ND
n	ND	ND
o	ND	ND
p	37.45	35.76
q	28.40	26.53
r	34.01	36.05
s	37.76	38.67
t	ND	37.18
u	37.55	37.28
v	ND	ND
w	38.87	38.87
x	ND	ND
y	ND	ND
z	ND	ND
a1	34.56	33.17

a ND, not detected.

### Outcome, interpretation, and troubleshooting analyses.

**FIG 3:**
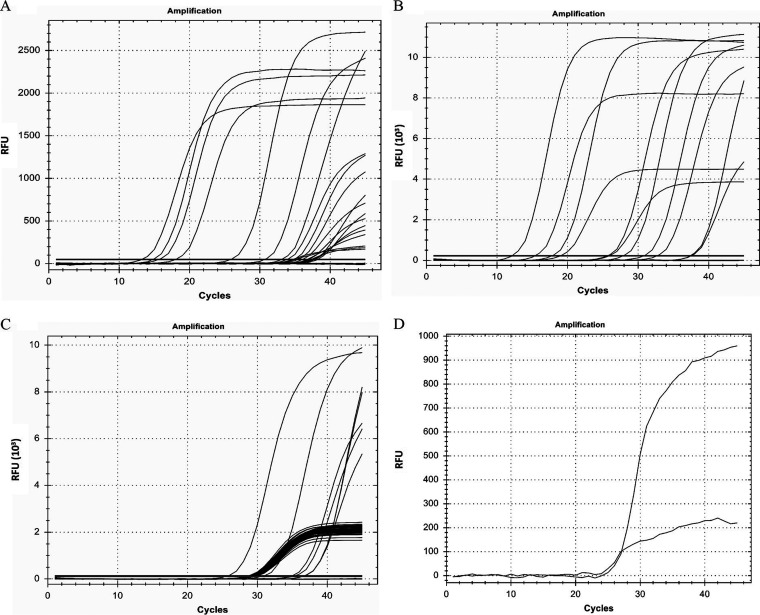
Results of the PCR analyses of reverse-transcribed RNA samples purified from the naso- and oropharyngeal swabs. (A) Representative results of initial PCRs run without optimization. (B) Representative results of their optimization. (C) Representative results of amplification of an internal control. (D) Representative results of an atypical curve detection. RFU, relative fluorescence units.

Figure 3 shows the representative curves obtained from PCR analyses of retrotranscribed RNAs purified from the NA samples of putatively infected individuals. The graphs displayed in the thermocyclers resulting from our first attempts at quantitative PCR showed nonuniform curves with several borderline low viral loads (see Fig. 3A). After carrying out an optimization of the enzymatic processes, most curves turned out more typically (Fig. 3B). Afterward, in our routing analyses, typical sigmoid amplification curves were either present (positive cases) or absent (negative cases) from the fluorescence channels corresponding to the viral genes tested, while the controls performed as expected (Fig. 3C). Depending on the amplification cycle where the sample initiated its curve, the *C_T_* value was assigned and the viral load inferred; the higher the former, the lower the latter. A common deviation from the expected sigmoid curves was somehow oddly shaped straight lines (see Fig. 3D). In this sense, having more than two probes for the analysis of the corresponding viral gene targets made the analysis difficult, because quite a few samples did not amplify all the targets, which resulted in delays in the analysis time to issue a result. Moreover, in other cases, individuals reporting clear COVID-19 symptoms but having negative results in the laboratory needed to be retested using double the amount of purified NAs, when usually they turned positive, although with a low viral load (high *C_T_* values).

## DISCUSSION

Realizing the unprecedented threat of the pandemic to our society, our primary objective as molecular biology diagnostic laboratories was to join the health professionals (the real heroes, bravely fighting in the trenches while poorly armed) to provide an opportune and reliable virus detection service. To do so, we quickly responded by implementing in our two laboratories (Monterrey and Mexico City) the SARS-CoV-2 molecular diagnosis service with the most confident results among the multitude of laboratories seizing the opportunity.

Essential to our plans was coordination with InDRE’s key personnel experts in both molecular biology and epidemiological surveillance. They are the authority responsible for setting the guidelines for laboratory work, biosafety while collecting, handling, and transporting samples, and protection of the personnel dealing with the samples, and they acted as consultants in case of doubt when interpreting the results and determining their clinical implications.

The first thing to do was to pass the comparative evaluation from InDRE, which included establishing the limits of detection (LODs) for the kits we chose for the detection of SARS-CoV-2 by RT-PCR (from the list published by InDRE), under the specific conditions of our laboratory. The second action item was to implement the service without putting our personnel at risk. To accomplish this and to reduce the risk of contagion, we redesigned the laboratory layouts to avoid interference between the flux paths of administrative personnel and the sample collectors, clients, and arriving samples. To further protect the personnel, we endowed the sample collectors and sample analyzers with reliable safety attire, implemented chemical disinfection protocols, and installed UV lamps in areas of higher contagion risk. The lack of contracted cases among laboratory operators as well as the lack of cross-contamination between samples was evidence that our implementation strategy was safe and successful, as described in Results.

We were conscious that the virus was lurking all over, from sampling, packaging, transportation, and unpackaging upon arrival in the laboratory and during the remaining handling processes before lysis of the biospecimens at the start of the purification step. Thus, precision and attention to detail were also essential to ensure traceability, speedy processing, preanalytical validation of materials, analytical accuracy, and opportune communication of the results to guide medical decisions.

Despite the commercial NA extraction kits’ claims to render material of high enough quality to guarantee good results, we routinely performed preanalytical analyses of the isolated NAs, derived either from a manual or automatic process. The UV spectrum profiles of the automatically isolated samples consistently showed low 260/230-nm ratio values, which is indicative of the possible presence of contaminants such as carbohydrates, peptides, or phenols in the sample (25). Although this supposedly low-quality indicator could be considered a disadvantage, automating the NA extraction process compensated by providing advantages such as less risk of contamination, better reproducibility, and reduced working time, thus resulting in better laboratory efficiency (26). One clear observation is that the manual method surpassed the automatic one in the parameter of yield.

In Table 2 and Fig. 2, we provide data from our comparison of the six commercially available RT-PCR kits for the detection of SARS-CoV-2 within our reach. From a selection of eight positives clinical samples (one with a high viral load and seven with low viral loads), just three kits were able to positively identify all.

Although we would agree that all the RT-PCR kits tested in this study can be used for routine diagnostics of symptomatic COVID-19 patients, the decision to work with one or another depends on the workload and capability of each laboratory. In our case, without a doubt, one of our main factors in choosing a particular kit was its sensitivity. Ironically, a challenge we faced was the higher sensitivity of our laboratory analyses over those of other commercial laboratories. Unsatisfied clients, generally involving asymptomatic (presymptomatic) workers at big companies who tested positive but had high *C_T_* values (in the upper 30s; very low viral loads), appealed to a laboratory from the competition, generally obtaining a negative result. Although a false positive entails less risk than a false negative, these companies pressed to not close their operations, dismissed our results, and did not listen to our advice to quarantine the potential infectious cases in their workforce. Moreover, such clients dismissed our explanation that the laboratory result is just an element, certainly a very important one, with the case study by their medical staff even more important to reach clinical (to pharmacologically treat or not) and occupational (to quarantine or not) decisions. Unfortunately, many of these companies either failed to take the results to their medical officer or simply had no such specialized professionals on their staff. In other words, confirmation of active infection should not rest only on the diagnostic laboratory result (27). This is even more true now that it is known that convalescent patients can continue to give a positive test result without being a source of contagion any longer, because rather than infectious virus, their bodies are eliminating the remains of the infection.

RT-PCR analysis proceeded in general with minimal complications. Internal controls, either for the samples or for the enzymatic reactions, were most valuable. To deal with atypical results with one type of kit, we repeated the analysis with another type of kit. When internal controls indicated potential inhibition, we repeated the RT-PCR with 2-fold diluted NA, which usually resolved the situation. And when all controls failed to give a signal but data from patients were suggestive of infection, we repeated the test, doubling the amount of purified NA.

In case of either internal doubts or external distrust in the results, we included a statement in the consent form signed by the clients to grant us the right to preserve the sample remnants for additional testing and future research. Additionally, for positive cases, we included in the result report a recommendation to seek medical advice, making clear to them that the laboratory results were only a piece of information, although of great value, to guide medical decisions. In turn, we pledged to protect all personal data from each of our clients.

It is important to clarify that the laboratory processes were not the only task that mattered; overseeing them through QC/QA to minimize mistakes, gathering patient information, and communicating positive cases to InDRE for national epidemiological surveillance were additional tasks that consumed even more of our time. This was especially true until we opted for automatizing all these tasks as much as possible, with the aid of informatics tools to build our laboratory information management system (which included barcoding of samples and laboratory order forms), and succeeded in implementing the ISO 9001-2015 system.

We want to believe that we achieved our goals efficiently, incentivized by the emergency and the one-in-a-lifetime opportunity to aid in the national fight against a pandemic of unprecedented dimensions (around half a million casualties officially recognized by the Mexican Government). Although initially we promised results in 48 to 72 working hours, soon we reduced the turnaround time to 24 h, subsequently to 8 h, and in pressing cases, to 6 h or even a bit less. Amid a scarcity of almost everything that put at risk our service and pledges to deliver results faster than any other laboratory, we were fortunate to have an ample network of generous contacts at local universities, representatives of laboratory supply manufacturers, and private clinical laboratories, who during the repeated crisis of supply shortages, helped us by lending us replacements for missing or failed pieces of equipment, materials, or reagents, so we never had to postpone our implementation plans.

Thus, we have achieved a real sense of accomplishment regarding our initial primary objective, i.e., to assist the clinicians caring for members of our society, at risk themselves of becoming victims of this feared enemy, with an opportune and reliable diagnosis that will allow them to treat the disease in its early phases, when it can be handled more efficiently pharmacologically.

### Conclusions.

We want to believe that we implemented our COVID-19 diagnostic laboratory services at both locations in a record time. The standardization and optimization of processes within a sanitary contingency was a great challenge that we met. Our previous experience in biosafety, molecular biology, and immunology, as well as working closely with the health authorities in charge of validating diagnostic technologies from abroad, were key to our success. Our conviction that any laboratory must adopt a QC/QA system, introduce process automatization as much as possible, and rely on informatics and the Internet to process and deliver results allowed us to offer companies, families, individuals, and health professionals the kind of accurate and opportune results that they needed to rely on to better control and deal with the ominous pandemic.

## MATERIALS AND METHODS

### Redirecting our laboratories as centers for molecular detection of SARS-CoV-2.

Before focusing most of our efforts on diagnosing COVID-19, our laboratories (Vitagenesis and Columbia Biotec) were keen on combating another national epidemic, that of human papillomavirus (especially its highly oncogenic types) and the cervical tumor that may result from not being able to clear the infection. After the approval of our new COVID-19 service (see below) by the government regulatory agency in charge of supervising diagnostic laboratories (the National Institute of Reference and Epidemiological Diagnosis, or InDRE—the acronym of its name in Spanish, Instituto Nacional de Diagnóstico de Referencia y Epidemiológica), our laboratories offered the test mainly to companies but also to the public, with a commitment to submitting a daily epidemiological and surveillance report of the processed samples to InDRE.

### Preparing the laboratories and their personnel to deal with SARS-CoV-2.

The laboratory areas, distribution of personnel, and fluxes of supplies and waste were reorganized to maintain unidirectionality and avoid intersecting paths. Special personnel were trained to deal with the potentially contagious samples. They wore special protection equipment, and a new laboratory area was put into operation for them to collect samples from patients (swabs of naso- and oropharyngeal exudates) and to receive specimens sent in category B packaging from or drawn on-site (usually by our trained personnel) at offices and manufacturing plants of the companies we served. After disinfection by the sampling personnel, the specimens were temporarily refrigerated while the laboratory personnel recovered them and moved them to a biosafety level 2 hood to take an aliquot for processing. They then divided the remains into aliquots to be stored temporarily (days) in the fridge (at the beginning of our service to the public, aliquots were submitted to InDRE for comparative evaluation) and more permanently later in the ultralow freezer (biobanking); this measure was taken in case reanalysis was needed or for research purposes (consented to by the clients).

### Sample handling and preservation.

The guidelines set by InDRE, which in turn were recommended by the WHO and the Centers for Disease Control (CDC) of the U.S. Government, were followed for adequate sampling and transportation of the naso- and oropharyngeal swabs. Aliquots (2.0 to 2.5 mL) of the laboratory-made viral transport medium (VTM) and/or commercial universal transport medium (UTM; catalog no. 330C; Copan, MBM, Jalisco, Mexico) were placed in sterile tubes kept under low-temperature conditions. Polystyrene swabs were mostly used. The samples were transported in triple packaging, duly labeled, and under cold conditions.

### Nucleic acid purification.

Aliquots of the naso- or oropharyngeal swab specimens were subjected to NA purification using either a manual procedure aided by the QIAamp viral RNA minikit (catalog no. 52906, Qiagen, Mexico City, Mexico) or the Daan viral RNA purification kits (catalog no. DA059; Daan Gene, Kabla, Mexico City, Mexico) or relying on automated techniques, using the Daan Smart 32 equipment along with the corresponding nucleic acid extraction kit (catalog no. DA0602; Daan Gene, Kabla).

### Preanalytical characterization of purified nucleic acids.

To guarantee patients an even more reliable result in the molecular detection of the SARS-CoV-2 virus, before proceeding to the molecular analysis (RT-PCR) of the NA purified from samples (specifically, the viral RNA regions of interest to be subjected to reverse transcription), samples were subjected to preanalytical qualification. This qualification consisted of spectrophotometer (NanoDrop 2000; Thermo Scientific, Mexico City, Mexico) readings to determine the purity (by the 260-nm/280-nm and 260-nm/230-nm ratios, as well as by the UV profiles) and concentration (to calculate yield). Deviation from the ideal purity values (260 nm/280 nm = 1.8 to 2.0; 260 nm/230 nm = 2.0 to 2.2; UV profiles lacking shoulders at wavelengths of 230 nm or 280 nm) can indicate contamination, either by aromatic compounds, salts, proteins, or carbohydrates, although such deviations do not necessarily affect the outcome of the RT-PCR analysis. These preanalytical characterization values, especially of the yield, were used to compare the performance of the automatic and manual extraction methods. A normalization test followed by a Wilcoxson comparison test was performed using GraphPad Prism version 9.4.0 for Windows (GraphPad Software, San Diego, CA, USA).

### Reserve transcription coupled with real-time PCR.

For this key NA test for SARS-CoV-2, we comparatively assessed most of the RT-PCR commercial kits approved by InDRE for diagnosis (Table 4). We choose to inspect not only their sensitivity and specificity but also their simplicity of use, time required for the steps involved, material consumed, and convenience and value of controls. Each kit was used according to the working instructions for either the Bio-Rad CFX96 C1000 Touch thermal cycler (Mexico City, Mexico), the Qiagen Rotor-Gene Q 5plex platform, or the Roche Cobas z 480 analyzer (Mexico City, Mexico).

**TABLE 4:** Quality features claimed by the manufacturer for the RT-PCR kits assayed

RT-PCR kit^*a*^	Target gene(s)	LOD^*b*^ (no. of copies/mL)	Sensitivity (%)	Specificity (%)
RIDA	E	4.3	100	100
Viasure	ORF1ab, N	20	95	100
Vircell	N, E	50	100	100
LightMix	N, RdRP, and E	10	100	100
1copy	E, RdRP	200	100	100
Logix Smart	RdRP	600	100	100

a RIDA gene kit (R-Biopharm AG, Darmstadt, Germany; catalog no. PG6815RUO); Viasure kit (CerTest Biotec, Zaragoza, Spain; catalog no. VS-NCO212L); Vircell SARS-CoV-2 RT-PCR kit (Granada, Spain; catalog no. RTPCR001); LightMix modular assay (Roche, Berlin, Germany; catalog no. 06754155001); 1copy qPCR multikit (1drop, Gyeonggi, South Korea; catalog no. M22MD100M), and Logix Smart kit (Co-Diagnostics, Salt Lake City, UT, USA; catalog no. COVID-K-001).

b LOD, limit of detection.

In addition to testing each of the six kits with numerous samples, we chose to compare them using nine specific samples, of which eight were positive and one negative. Of the positive samples selected, seven had low viral loads and one high viral load. An additional 27 samples were comparatively analyzed using the Logix Smart and the RIDA kits.

Although we preferred to alternate between the RIDA, Viasure, and Vircell kits, especially when the workload was high, we preferred the first two not only for their better detection limit but also because of other factors, such as the number of supplies needed and the time spent assembling reactions (see Table 2).

### Results and troubleshooting interpretation.

For the preparation of the reports of the RT-PCR analysis results, the presence or absence of a typical sigmoid amplification curve was the decisive factor in declaring a test positive or negative, respectively, as long as the controls performed as expected. In addition to judging the results of each kit, each kit had a different interpretation depending on whether the reaction contained more than two targets for detection of the virus. Moreover, the apparent physical conditions of the individual being tested were also considered—for example, if on the questionnaire required prior to testing, they checked the boxes for suspicious symptoms, history of exposure to a confirmed infected contact, recent international trip, and/or if symptomatic, whether they were starting or ending the clinical phase. The response to this latter question was key to judging if a low viral load detected corresponded to an infection just beginning or ending. Cycle threshold (*C_T_*) values below 40 were considered positive; values above this cutoff (or if there was no amplification at all) were considered negative. Invalid samples, considered as such when errors occurred in the execution of the purification and/or the PCR, reflected in failure of the amplification of the internal control(s), were always reprocessed.

### Quality control/quality assurance and automation.

We hired a consultancy to audit our general laboratory processes for implementation of ISO 9001/2015; using internal and external information technology (IT) support, we managed to make the laboratory workflow a better one.
